# N-OH-AABP Modifications in Human DNA May Lead to Auto-Antibodies in Bladder Cancer Subjects

**DOI:** 10.3390/diagnostics12020337

**Published:** 2022-01-28

**Authors:** Uzma Shahab, Safia Habib, Ahmad Alsulimani, Qurain Turki Alshammari, Abdulrahman A. Alatar, Shafiul Haque, Moin Uddin, Saheem Ahmad

**Affiliations:** 1 Department of Biochemistry, J.N. Medical College, Aligarh Muslim University, Aligarh 202002, India; uzmashahab@gmail.com (U.S.); saf_h75@yahoo.co.in (S.H.); 2 Medical Laboratory Technology Department, College of Applied Medical Sciences, Jazan University, Jazan 45142, Saudi Arabia; aalsulimani@jazanu.edu.sa; 3 Diagnostic Radiology Department, College of Applied medical Sciences, University of Hail, Hail 2440, Saudi Arabia; g.algrain@uoh.edu.sa; 4 Department of Botany & Microbiology, College of Science, King Saud University, P.O. Box 2455, Riyadh 11451, Saudi Arabia; aalatar@ksu.edu.sa; 5 Research and Scientific Studies Unit, College of Nursing and Allied Health Sciences, Jazan University, Jazan 45142, Saudi Arabia; shafiul.haque@hotmail.com; 6 Bursa Uludağ University Faculty of Medicine, Görükle Campus, Nilüfer, Bursa 16059, Turkey; 7 Department of Medical Laboratory Sciences, College of Applied medical Sciences, University of Hail, Hail 2440, Saudi Arabia

**Keywords:** 4-aminobiphenyl (4-ABP), N-hydroxy-Acetyl 4-Aminobiphenyl (*N*-OH-AABP), bladder cancer, DNA, carcinogen

## Abstract

4-Aminobiphenyl (4-ABP) and other related arylamines have emerged to be responsible for human urinary bladder tumors and cancers. Hemoglobin-ABP adducts have been recognized in the blood of smokers, and it builds up in the circulatory system over the period of years that might lead to a bladder tumor. N-hydroxy-Acetyl 4-Aminobiphenyl (N-OH-AABP) is one of the reactive forms of 4-ABP which has a potential to initiate tumor growth and causes cancer rapidly. In the present study, commercially available human DNA was modified by N-OH-AABP, and its modifications were analyzed biophysically from fluorescence spectroscopy and thermal denaturation studies. Further, Sera and IgG from bladder cancer patients’ blood were assessed for affinity to native and N-OH-AABP modified human DNA using ELISA. The study showed N-OH-AABP caused damage in the structure of the DNA macromolecule and the perturbations resulting from damage leads to change in the Tm of the DNA molecule. Bladder cancer auto-antibodies, particularly in smoker group, showed preferential binding to N-OH-AABP modified human DNA. This study shows that N-OH-AABP modified DNA could be an antigenic stimulus for the generation of autoantibodies in the sera of bladder cancer patients.

## 1. Introduction

Aromatic amines are among the first chemical carcinogens that have been implicated in human cancers [1]. Ample evidence from both epidemiological studies and animal models has firmly established that arylamines, particularly, 4-aminobiphenyl (4-ABP), are the major culprits in bladder cancer related to occupational exposures [2]. 4-ABP is also known to be an environmental contaminant generated largely from cigarette smoke, as well as the combustion of fossil fuels and from coal and rubber industries [3,4]. It has been revealed to be a foremost etiological driving force of human bladder cancer, and also to be a strong urinary bladder carcinogen in experimental animals. There is also a study in which hemoglobin and the DNA isolated from the bladder of smokers potentially accuses 4-ABP in the etiology of bladder cancer. The International Agency for Research on Cancer (IARC) has classified 4-ABP as a Group I carcinogen, resulting in the cessation of commercial production and usage of 4-ABP. However, the aromatic amine 4-aminobiphenyl still continues to be an environmental and occupational contaminant, since it is generated mainly from cigarette smoke.

**Scheme 1:**
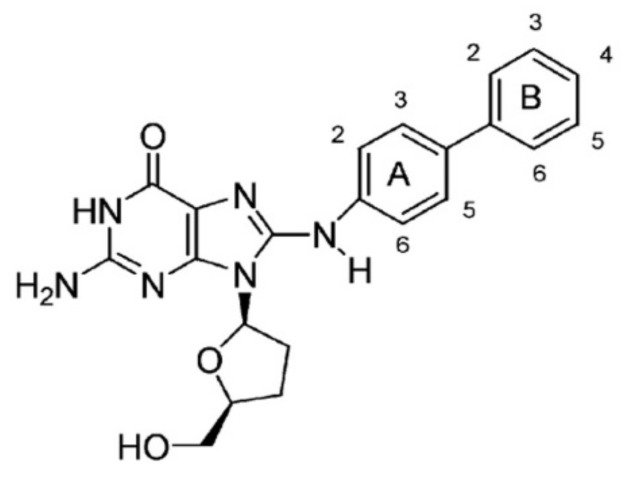
Structure of dG-C8-4-ABP adduct.

The activation of arylamines in general involves *N*-oxidation by specific cytochrome P450 (CYP1A2) in hepatic tissues [5], followed by conjugation of the *N*-hydroxyl with acetate, sulfate, or glucuronate [6]. In bladder carcinogenesis, the *N*-glucuronides formed by hepatic metabolism are postulated to be transported to the urinary bladder, where they are hydrolyzed to the *N*-hydroxy derivatives. The pro-carcinogens, *N*-hydroxy metabolites, namely *N*-hydroxy-4-acetylaminobiphenyl (*N*-OH-AABP) and *N*-hydroxy-4-aminobiphenyl (*N*-OH-ABP), could be activated by the enzyme systems that are present in the urothelium. The *N*-hydroxy arylamines can then be converted to aryl nitrenium ions through the putative intermediate. These electrophilic nitrenium ions interact with DNA to form covalent DNA adducts (dG-C8-4-ABP), thereby exerting their genotoxic effects [7]. The predominant DNA adduct, dG-C-8-4-ABP adduct is shown in Scheme 1. 4-ABP–DNA adducts have been detected in the exfoliated urothelial cells of cigarette smokers and their levels correlate with the levels of 4-aminobiphenyl–hemoglobin adducts in the same subjects [8]. The genotoxic effect of 4-ABP and its derivatives on human DNA, as well as its implications on cancer, has been reported by us and others [9,10,11]. There are no studies which focused on the auto-antibody detection against the *N*-OH-AABP modified DNA in the sera of cancer patients. Therefore, the present study reports the biophysical changes induced in human DNA on exposure to a carcinogen, *N*-OH-AABP. The study also compares the binding ability of smokers’ and non-smokers’ groups of auto-antibodies with *N*-OH-AABP modified DNA using direct binding ELISA and band shift assay.

## 2. Materials and Methods

### 2.1. Serum Sample Collection

The investigation was performed for 100 human subjects in which 40 cancer sera were from the smokers’ group, and 40 cancers were from the non-smokers’ group. The samples of 20 healthy subjects were used as negative control. The patients visiting the OPD/IPD in JNMCH, Aligarh, India, beforehand were analyzed and set apart as control and bladder cancer subjects for the present investigation. The examination has been completed as per the declaration of Helsinki. For this study, 5 mL of blood samples were acquired from each healthy and cancer volunteer after the informed consent. None of the cancer patients had other autoimmune diseases. The serum samples were collected in a glass test tube and left to clot for one hour at room temperature. The serum was then separated by centrifugation at 3000 rpm for 10 min. Serum samples were heated at 56 °C for 30 min to inactivate complement proteins and stored in aliquots at −20 °C, with 0.1% sodium azide added as preservative.

### 2.2. Purification of Human Placental DNA

Human placental DNA was purified to be free of proteins and single stranded areas with slight alterations. Briefly, DNA (2 mg/mL) was dissolved in 0.1 mM sodium citrate buffer, pH 7.3, and separated with an equivalent volume of chloroform-isoamyl alcohol (24:1) in a stoppered compartment and shaken for 1 h. The fluid layer containing DNA was isolated from organic layer and re-extracted with chloroform-isoamyl alcohol mixture. DNA was precipitated with two volumes of chilled absolute ethanol and gathered on a glass bar. In the wake of drying in air, the DNA was dissolved in acetic acid derivation buffer (30 mM sodium acetate containing 30 mM zinc chloride, pH 5.0) and treated with nuclease S1 (150 units/mg DNA) at 37 °C for 30 min to expel single stranded areas. The reaction was ended by adding one-tenth volume of 200 mM ethylene diamine tetra acid (EDTA), pH 8.0. Nuclease S1 treated DNA was separated twice with chloroform-isoamyl alcohol and lastly precipitated with two volumes of chilled ethanol. The precipitate was dissolved in the required buffer. The purity of DNA was affirmed by A260/A280 ration, which was in the scale of 1.8–2.0.

### 2.3. Modification of Human Placental DNA

10 μM of human placental DNA was modified with *N*-OH-AABP at different concentrations, as described previously [12]. Briefly, human placental DNA (10 μM) from Sigma (St. Louis, MO, USA) was modified by incubating at 37 °C for 24 h with varying concentrations (0.378, 0.757, 1.136, and 1.515 mM) of N-OH-AABP (Midwest Research Institute, Kansas City, Kansas) in dimethyl sulfoxide (DMSO). The unbound constituents were removed by extensive dialysis against sodium phosphate buffer, pH 7.4.

### 2.4. Fluorescence Spectroscopy

Fluorescence emission spectral investigation of native and N-OH-AABP altered human DNA tests (5 μg/mL) was undertaken in the wavelength range of 300–700 nm, utilizing a quartz cuvette on a Shimadzu RF-5301 spectrofluorometer at an excitation wavelength of 325 nm. Ethidium bromide (2.5 μg/mL) was utilized as an external chromophore to test for the DNA damage caused by the cancer-causing agent N-OH-AABP. Due to its unique structure, it can easily intercalate into DNA strand; in this way, it is ordinarily utilized as nucleic acid fluorescent tag.

### 2.5. Thermal Denaturation Studies

Thermal denaturation analysis of DNA was performed to discover the thermal steadiness of DNA constituent to change [13]. The midpoint melting temperature (Tm) of native and altered DNA tests was dictated by subjecting the samples to thermal denaturation on Shimadzu UV-1700 Spectrophotometer furnished with a thermo-software and controller unit. All of the samples experienced temperature range from 30 °C to 95 °C at a rate of 1.0 °C/min. The adjustment in absorbance at 260 nm was recorded with increasing temperatures.

Percentage of denaturation was calculated as per the following equation:

Percent denaturation = (A_T_ − A_30_/A_max_ − A_30_) × 100

where, A_T_ = absorbance at a temperature T °C, A_max_ = final maximum absorbance on the completion of denaturation (95 °C), and A_30_ = initial absorbance at 30 °C.

### 2.6. Direct Binding ELISA

Direct binding ELISA was carried out as described earlier [14]. Immunoglobulin G (IgG) was affinity purified from pre-immune and immune sera on a protein A-Agarose column [15,16].

### 2.7. Competition ELISA

The specific binding characteristics of antibodies were ascertained through a competitive binding assay. Competition ELISA was performed as described by Ahmad et al. in 2020 [17].

### 2.8. Band Shift Assay

The band shift test was executed as described previously. Briefly, antigen-antibody specificity was additionally affirmed by the gel retardation test (band shift assay). A consistent measure of DNA antigen (1 µg) was blended with varying amounts of IgG, and incubated for 2 h at 37 °C and overnight at 4 °C. Toward the end of the incubation, a one-tenth volume of test buffer was added to antigen-antibody complex and electrophoresed on 1% agarose gel in TAE buffer (pH 7.9) for 2 h at 30 mA current. The gels were stained with ethidium bromide (0.5 mg/mL) and imaged under UV light and captured.

## 3. Results and Discussions

### 3.1. Fluorescence Spectroscopy

**Figure 1:**
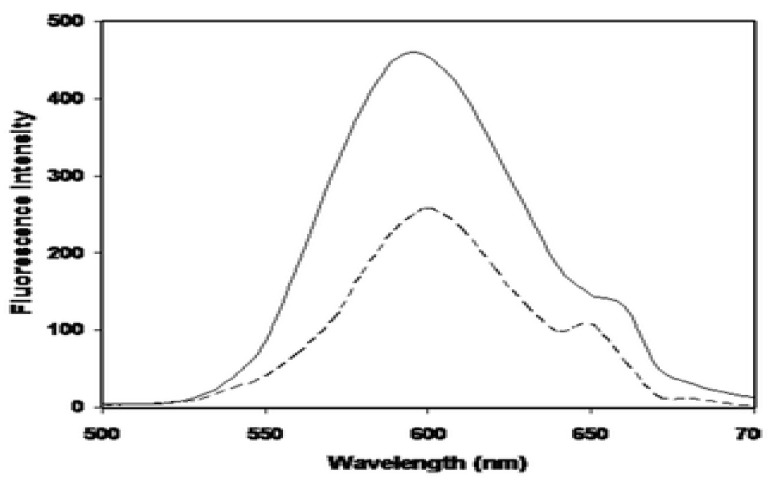
Fluorescence emission spectra of native human DNA (–––) and modified human DNA with 1.515 mM N-OH-AABP (–). The fluorescence scan was performed in triplicate. The plot was created by taking the average of three scans. The modification was statistically significant as compared to the control (*p* < 0.005). Reprinted with permission from Ref. [12]. Copyright 2013 PLoS ONE.

Both native and modified human DNA were incubated with ethidium bromide for 30 min, and the emission profile was recorded using an excitation wavelength (325 nm) of ethidium bromide. The emission profile of DNA, both native and *N*-OH-AABP modified human DNA, was recorded at 600 nm. As per our previously reported work, we found a decrease in 43.17% fluorescence intensity in the case of modified human DNA as compared to its native form, signifying perturbations in DNA double helical structure as a result of the *N*-OH-AABP modification (Figure 1) [12]. A decrease in the fluorescence intensity of the EtBr-*N*-OH-AABP-modified human DNA compared to EtBr-DNA in the fluorescence emission spectra confirms the destruction of structural integrity of DNA and generation of strand breaks, which result in poor intercalation of EtBr in the modified form of DNA [18].

### 3.2. Thermal Denaturation

**Figure 2:**
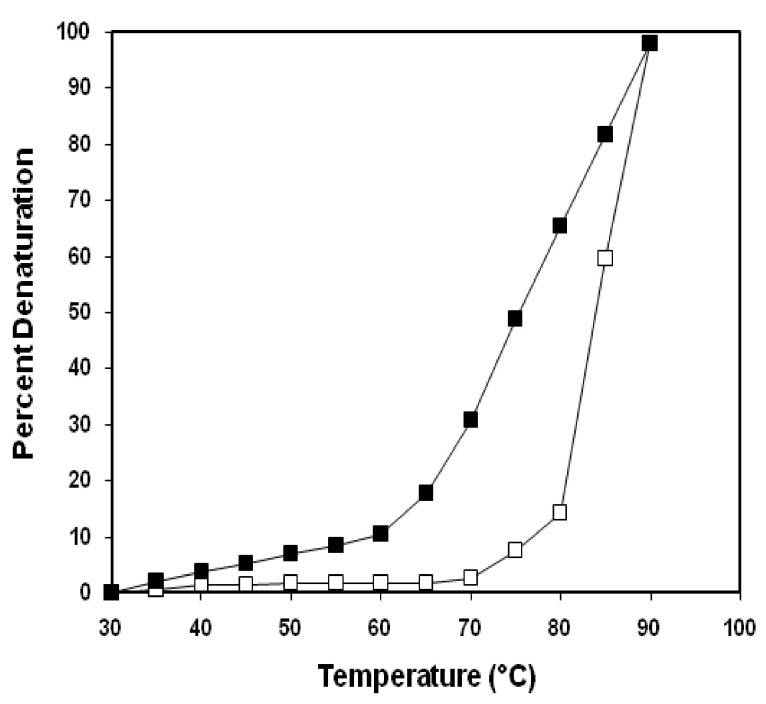
Thermal melting profile of native human DNA (□) and *N*-OH-AABP-modified human DNA (■). The thermal denaturation experiment was performed in triplicate. The drop in Tm is statistically significant for modified human DNA as compared to the native human DNA.

The melting temperature, T_m_, of native DNA was found to be 83.6 °C, while in the case of modified DNA, it was recorded to be 74 °C (Figure 2). The melting temperature study shows a drop of 9.4 °C for the *N*-OH-AABP modified human DNA, as compared to the control. A drop in 9.4 °C is a significant decrease in the T_m_ value of modified DNA, as compared to its unmodified native form. Upon modification by *N*-OH-AABP, DNA showed susceptibility to a rise in temperature when subjected to thermal denaturation studies. The decrease in Tm, therefore, points towards the destabilization of base stacking and hydrogen bonding and consequent helix disruption [19].

### 3.3. Binding of Serum Antibodies in Bladder Cancer Patients with Smoker and Non-Smoker Groups

**Figure 3:**
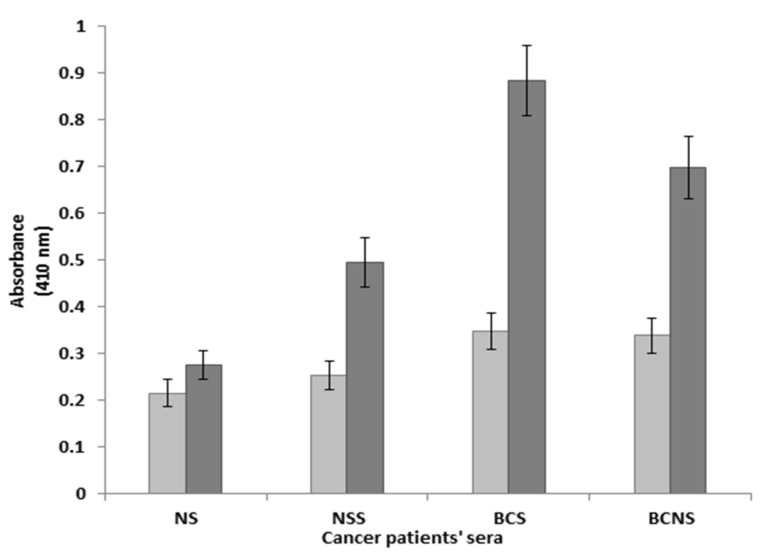
Direct binding ELISA of serum autoantibodies from bladder cancer patients of the smoker group (BCS) and the non-smoker group (BCNS) to native (■) and *N*-OH-AABP-modified human DNA (■). Normal human sera (NS) along with normal human smoker sera (NSS) served as a negative control. The histogram representing NS and NSS show mean absorbance values SD for binding of NS (*n* = 20). The plate was coated with *N*-OH-AABP-modified human DNA (2.5 μg/mL). The microtiter plates were coated with *N*-OH-AABP-modified human DNA (2.5 g/mL). The increase in autoantibodies in BCS and BCNS as compared to the NS and NSS is statistically highly significant. Both are statistically significant to the extent of more than 95%.

A total of 80 serum tests were examined for auto-antibody recognition. Overall, 40 samples were from the patients with a background marked by smoking, while the other 40 samples came from non-smokers. Sera from age and sex-matched normal healthy people (*n* = 20) served as a control (Figure 3). In the smoker group of bladder cancer patients, higher binding with N-OH-AABP-modified DNA was seen in all samples. However, for further studies, just those examples were chosen in which the binding with N-OH-AABP-DNA was more than twofold the binding with the native DNA. Of the aggregate of 40 samples from the smoker group, 29 samples (72.5%) indicated higher binding and were chosen for further studies. This rate is enormous, and is likely a biomarker in case of bladder cancer.

On other hand, among the non-smoker group, 16 samples (40%) showed more than double binding with *N*-OH-AABP-DNA, and hence were chosen for further studies to authenticate the presence of auto-antibodies. The difference of 32.5% was the significant difference between the smoker and the non-smoker groups for patients with the same disease, i.e., bladder cancer patients. This provides further opportunities for the cause and effect of smokers over the statistics of the bladder cancer patients. First, inference can be made from the direct binding results: the DNA molecules might be on continuous stress among the smoker group; secondly, the carcinogen, *N*-OH-AABP, which is also a constituent of cigarette smoke and automobile discharge might damage the human DNA and present unique epitope [20], wherein the similar epitope might be missing in the DNA of non-smokers. Still, this is debatable in cancer biology to probe for the prevalence/presence of auto-antibodies against the modified DNA from carcinogens, which can be used one of the biomarkers of future for the early detection and/or prognosis of the disease.

### 3.4. Auto-Antibodies against N-OH-AABP Modified Human DNA as a Diagnostic Marker for Bladder Cancer in Smoker and Non-Smoker Groups

**Figure 4:**
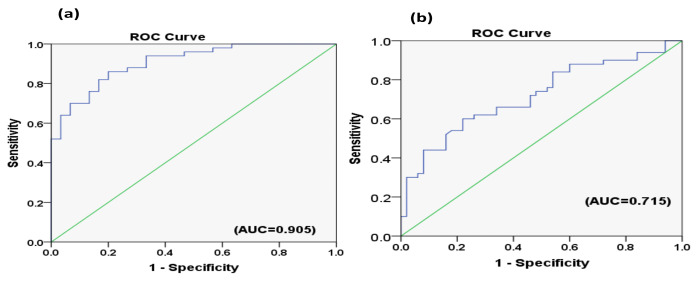
ROC curve analysis. (**a**) Smoker group: auto-antibodies against N-OH-AABP-modified human DNA can distinguish bladder cancer patients from controls (AUC = 0.905). (**b**) Non-smoker group: the relatively low presence of auto-antibodies against N-OH-AABP-modified human DNA in the non-smoker group of bladder cancer patients (AUC = 0.715). ROC, receiver operating characteristic; AUC, area under the curve.

In order to confirm the presence or prevalence of auto-antibodies against N-OH-AABP-modified human DNA as diagnostic marker in the sera of bladder cancer subjects, a receiver operating characteristic (ROC) and area under the curve (AUC) was established. where AUC 0.905 with 85% sensitivity and 80% specificity in the smoker group of bladder cancer patients compared to adjacent controls (Figure 4a) was observed. Moreover, the AUC 0.715 with 68% sensitivity and 62% specificity in non-smokers (Figure 4b) was observed with respect to their adjacent control. The results showed that auto-antibodies against N-OH-AABP-modified human DNA in the smoker group may be used as a diagnostic biomarker, since they have the ability to discriminate bladder cancer patients from their controls.

### 3.5. Competitive Inhibition ELISA

The binding specificity of the isolated IgG towards native and *N*-OH-AABP-modified human DNA was evaluated by inhibition ELISA. The observed antibody (IgG) inhibition for smokers and non-smokers ranged from 58.4 to 74.4% and 47.1 to 55.1%, respectively, when modified human DNA was employed as an inhibitor, while with the native human DNA it varied from 27.6 to 33.8% and 22.2 to 29.4%, respectively. The mean of inhibitions with *N*-OH-AABP-modified human DNA as an inhibitor was calculated to be 65.25 ± 4.9% and 44.13 ± 5.3% in the smoker and non-smoker groups, respectively. Table 1 and Table 2 summarize the inhibition data of isolated IgGs from bladder cancer patients in the smoker group and non-smoker groups, respectively.

The mean ± SD of maximum percentage inhibition in the smoker group at 20 mg/mL was 65.25 ± 4.9, as compared to the native human DNA which is 30.96 ± 1.9. The higher maximum percentage inhibition was statistically significant in modified human DNA, as compared to the native DNA. The *p* value was found to be * *p* < 0.005.

The microtiter plates were coated with *N*-OH-AABP-modified human DNA (2.5 g/mL). The mean ± SD of maximum percentage of inhibition in the non-smoker group at 20 mg/mL was 51.19 ± 3.0, as compared to the native human DNA, which was 29.11 ± 3.5. The higher maximum percentage inhibition was statistically significant in the modified human DNA as compared to the native DNA. The *p* value was found to be * *p* < 0.01.

### 3.6. Band Shift Assay

**Figure 5:**
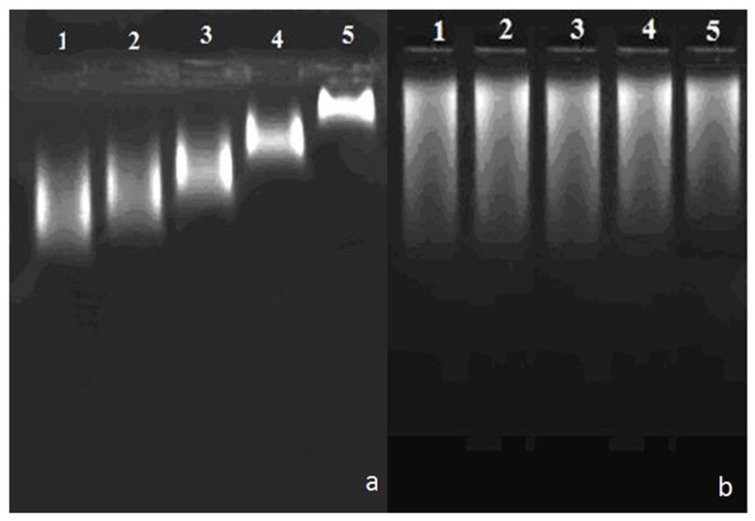
Band shift assay of IgG from bladder cancer patients (smoker group) using (**a**) modified human DNA and (**b**) native human DNA. Electrophoresis was carried out on 0.8% agarose gel for 2 h at 30 mA.

The auto-antibodies from the smoker group indicated more specific binding; they were subsequently utilized for a band shift assay to visually recognize the interaction of native or N-OH-AABP-modified human DNA with the purified IgG from bladder cancer patients. The immune complex of N-OH-AABP DNA with the purified IgG caused a relative increment in the gradual mobility in retardation and gradual increase in the band intensity near the wells in agarose gel electrophoresis. In any case, native DNA did not exhibit any noticeable retardation in mobility, which shows that the immune complex formation under identical conditions did not occur with native DNA [21] (Figure 5a,b). This prompts us to conclude that N-OH-AABP creates neo-epitopes on the DNA macromolecule that are perceived as ‘alien’ or non-self by the immune system, bringing about autoantibody generation in bladder cancer patients.

## 4. Conclusions

The present study discusses the modification of human DNA brought about by a carcinogen, N-OH-AABP. The clinical study focused on the presence or prevalence of autoantibodies in the sera of bladder cancer patients against native and N-OH-AABP modified human DNA in the smoker and non-smoker groups. Our results indicate there is a high prevalence of auto-antibodies in case of the smokers group, as compared to the non-smoker groups in the sera of bladder cancer patients. We believe that the antibodies which are generated as a response of modified human DNA are highly prevalent and specific, as evident from the competitive inhibition ELISA and band shift assays. Similar results were produced in our previous studies, in which antibodies were raised against modified proteins and DNA macromolecules [22,23,24,25]. The prevalence of auto-antibodies in direct binding ELISA was found to be highly significant in modified human DNA as compared to the native human DNA in the sera of bladder cancer patients in both the smoker and non-smoker groups. The auto-antibodies against modified human DNA in the sera of smoker and non-smoker group was statistically significant to the extent of *p* < 0.001. Similarly, the maximum percentage inhibition was highest in the cancer sera of the smoker group, which was recorded to be *p* < 0.005 as compared to the non-smoker group, *p* < 0.01. The preferential binding of antibodies from bladder cancer patients to N-OH-AABP-modified DNA indicates better recognition of epitopes on the modified DNA by these antibodies, implicating the role of N-OH-AABP-modified DNA in the generation of autoantibodies in bladder cancer patients. The neoepitopes on N-OH-AABP-modified DNA result in the formation of auto-antibodies in the sera of smoker and non-smoker groups in bladder cancer subjects. This might be used as a biomarker for the early detection of bladder cancer in the smoking population. To further confirm the prevalence of auto-antibodies against N-OH-AABP-modified human DNA as diagnostic marker characteristic in the sera of bladder cancer subjects, a receiver operating characteristic (ROC) and area under the curve (AUC) implicates that auto-antibodies against N-OH-AABP-modified human DNA in the smoker group may be used as a diagnostic biomarker since it has the ability to discriminate bladder cancer patients from their controls as well as from non-smoker groups as well. However, the study was restricted to the identification of auto-antibodies against N-OH-AABP-modified human DNA. Our future plan will be to correlate IgG antibodies with different grades or stages of bladder cancer subjects.

## Figures and Tables

**Table 1:** Competitive inhibition data of IgG from bladder cancer patients (smoker group).

	Maximum Percent Inhibition at 20 g/mL	
Sera No.	Native Human DNA	*N*-OH-AABP Modified Human DNA
01	27.6	67.9
05	28.7	60.8
06	32.3	61.9
08	32.6	63.3
09	30.8	64.0
10	31.4	61.5
12	33.0	66.1
15	31.4	68.3
19	33.8	59.0
20	28.6	65.8
28	33.0	58.4
29	29.3	74.4
30	28.8	58.6
33	30.4	69.5
34	33.0	66.2
36	32.7	73.8
38	28.4	72.0
39	31.6	63.0
**Mean ± SD**	**30.96 ± 1.9**	**65.25 ± 4.9**

**Table 2:** Competitive inhibition data of IgG from non-smoker group of bladder cancer patients.

	Maximum Percent Inhibition at 20 g/mL	
Sera No.	Native Human DNA	*N*-OH-AABP Modified Human DNA
01	29.7	49.8
04	26.2	56.2
10	33.3	49.1
13	27.8	48.2
14	25.1	53.4
19	28.7	47.1
21	32.0	49.6
33	29.3	55.1
35	24.2	50.8
40	34.8	52.6
**Mean ± SD**	**29.11 ± 3.5**	**51.19 ± 3.0**
